# Single-sex infection with female *Schistosoma mansoni* cercariae mitigates hepatic fibrosis after secondary infection

**DOI:** 10.1371/journal.pntd.0005595

**Published:** 2017-05-19

**Authors:** Nicole Koslowski, Martina Sombetzki, Micha Loebermann, Robby Engelmann, Niels Grabow, Christoph H. Österreicher, Michael Trauner, Brigitte Mueller-Hilke, Emil C. Reisinger

**Affiliations:** 1 Department of Tropical Medicine and Infectious Diseases, University Medical Center, Rostock, Germany; 2 Institute of Immunology, University Medical Center, Rostock, Germany; 3 Institute for Biomedical Engineering, University Medical Center, Rostock, Germany; 4 Institute of Pharmacology Center for Physiology and Pharmacology Medical University, Vienna, Austria; 5 Hans Popper Laboratory of Molecular Hepatology, Department of Internal Medicine III, Medical University, Vienna, Austria; University of Manchester, UNITED KINGDOM

## Abstract

**Background:**

Infection with *Schistosoma spp*. affects more than 258 million people worldwide. Current treatment strategies are mainly based on the anthelmintic Praziquantel, which is effective against adult worms but neither prevents re-infection nor cures severe liver damage. The best long-term strategy to control schistosomiasis may be to develop an immunization. Therefore, we designed a two-step *Schistosoma mansoni* infection model to study the immune-stimulating effect of a primary infection with either male or female cercariae, measured on the basis of TH1/TH2-response, granuloma size and hepatic fibrosis after a secondary bisexual *S*. *mansoni* challenge.

**Methodology/Principle findings:**

As a first step, mice were infected with exclusively female, exclusively male, or a mixture of male and female *S*. *mansoni* cercariae. 11 weeks later they were secondarily infected with male and female *S*. *mansoni* cercariae. At week 19, infection burden, granuloma size, collagen deposition, serum cytokine profiles and the expression of inflammatory genes were analyzed. Mice initially infected with female *S*. *mansoni* cercariae displayed smaller hepatic granulomas, livers and spleens, less hepatic fibrosis and higher expression of Ctla4. In contrast, a prior infection with male or male and female *S*. *mansoni* did not mitigate disease progression after a bisexual challenge.

**Conclusions/Significance:**

Our findings provide evidence that an immunization against *S*. *mansoni* is achievable by exploiting gender-specific differences between schistosomes.

## Author summary

Schistosomiasis remains a major cause of morbidity and mortality, and in the tropics and subtropics in particular, infection rates are high. The efficacy of anthelmintic therapy is limited since it has no effect on immature parasite stages and does not prevent re-infection. The root cause of disease is a granulomatous hypersensitivity reaction to parasite eggs entrapped within the intestinal wall and small liver sinusoids. This reaction is mainly caused by CD4^+^ T cells of the subtype 2 and alternatively activated macrophages. As a repair response it suppresses inflammation but results in hepatic fibrosis (e.g. Symmer's pipe stem fibrosis), portal hypertension and its clinical sequelae, ascites and esophageal varices. We demonstrated herein that a primary infection with female *S*. *mansoni* cercariae leads to the suppression of TH2-mediated granuloma size, hepatic fibrosis and disease progression in bisexually challenged mice. This protection is associated with Retnla- and Ctla4-mediated TH2 suppression but not with a reduction in parasite load. Our findings provide evidence that protection against egg-induced granulomatous hyperreactivity is achievable by exploiting gender-specific differences between schistosomes.

## Introduction

The blood flukes of the genus *Schistosoma spp*. are among the world's most prevalent human helminthic parasites. According to the WHO over 258 million people are currently receiving preventive therapy, mostly to avoid severe long-term liver damage [1]. During their life-span of up to 15 years, schistosomes produce a myriad of tissue-damaging eggs [2]. Entrapped within the intestinal wall and small liver sinusoids they provoke an inflammatory, granulomatous reaction that is mainly caused by CD4^+^ T cells of the subtype 2 and alternatively activated macrophages [3]. This repair response suppresses initial TH1 inflammation but results in hepatic fibrosis (e.g. Symmer's pipe stem fibrosis), portal hypertension and its clinical sequelae, ascites and esophageal varices [4].

*S*. *mansoni* infection triggers a transient T-helper-1 (TH1) cell reaction mediated by proinflammatory cytokines such as IFN-γ, TNF-α, IL-12, and iNOS. Following the onset of egg production, the inflammatory TH1-response shifts towards a profibrotic TH2-response mediated by IL-4 and IL-13 [5–7]. However, this TH1/TH2 dogma is not as stringent as formerly supposed, since it has been shown that isolated *S*. *mansoni* eggs and soluble egg antigens suffice to induce a TH2-response in mice [7–9]. Moreover, the discovery of IL-4-responsive macrophages before the onset of egg production indicates the presence of a TH2-dominant milieu from early on [10].

Loss of worm integrity leads to a strong release of antigens and thus results in a certain resistance to re-infection. This protection can be hastened factitiously by the killing of adult worms with Praziquantel, but also occurs when worms die naturally [11, 12].

A number of studies have looked into the generation of immunity against adult schistosomes using sterile, unisexual infection models. It is known that soluble *S*. *mansoni* worm antigens (SWA) sensitize mice to granuloma formation, and when injected into the tail vein of naive mice, parasite eggs form perioval granulomas that are smaller in size and which differ in cellular composition to the granulomas found in mice pre-infected with either male or female cercariae [13]. In the studies in question, single-sex infection with male Schistosoma cercariae led to pronounced organ changes (increased liver and spleen weight), delayed-type hypersensitivity and higher numbers of peripheral blood cells in mice [14], whereas single-sex infection with female Schistosoma cercariae increased antibody response in baboons [15]. In contrast, cell-mediated immunity was observed in splenocytes isolated from mice infected with both sexes of cercariae compared to single sex infected mice [16]. However, protection after unisexual infection was not achievable when reinfection was performed more than 6 weeks later [17].

These discrepancies led us to revisit the issue of unisexual infection, placing special emphasis on the expression of proinflammatory and profibrotic markers. To this end we designed a two-step *Schistosoma mansoni* infection model measuring TH1/TH2-response, granuloma size and hepatic fibrosis.

## Methods

### Ethics statement

All experiments were performed according to German animal protection regulations and approved by the local committee on animal care and use (7221.3–1.1-008/13).

### *Schistosoma mansoni* infection

Different stages of *Schistosoma mansoni* (Belo Horizonte strain) were kept using *Biomphalaria glabrata* freshwater snails (*B*. *glabrata*, Brazilian strain) as intermediate hosts and 6–8 week-old female NMRI mice as definitive hosts, as previously described. In brief, cercariae were obtained by using light exposure to trigger mass shedding, and the number of cercariae/ml was determined using a conventional light microscope (100-fold magnification). Mice were kept on a 12:12 hour light/dark cycle and given standard mouse chow (SSNIFF, Soest, Netherlands) and water *ad libitum*. *B*. *glabrata* were kept in aquarium water at 25°C on a lettuce diet [18].

### Two-step *Schistosoma mansoni* infection model

Separated *B*. *glabrata* were exposed to single *S*. *mansoni* miracidia to obtain either female or male cercariae from each snail 6 weeks later [19]. The sex of the cercariae was determined by DNA amplification of sex-related chromosome segments using female-specific W1 and W2 primers and male/female specific Sm23 primers as a positive control (Table 1).

**Table 1:** Primers used to determine male and female *S*. *mansoni* cercariae[19,20–22].

Gene	Forward Primer	Reverse Primer	Access. No.
**W1**	5´CAACACAGTGAAATTCTTCC 3´	5´GAATTCACCACTCGACATTC 3´	J04665.1
**W2**	5´TTGCTGATGTGCAGTTTGCC 3´	5´TCTTCCGAGTATGATGCAGG3´	U10109.1
**Sm23**	5´TGGGTACTGGTATGCGTTGT 3´	5´CAGCATGCAGACGTTTTCCT 3´	M34453.1

**Fig 1:**
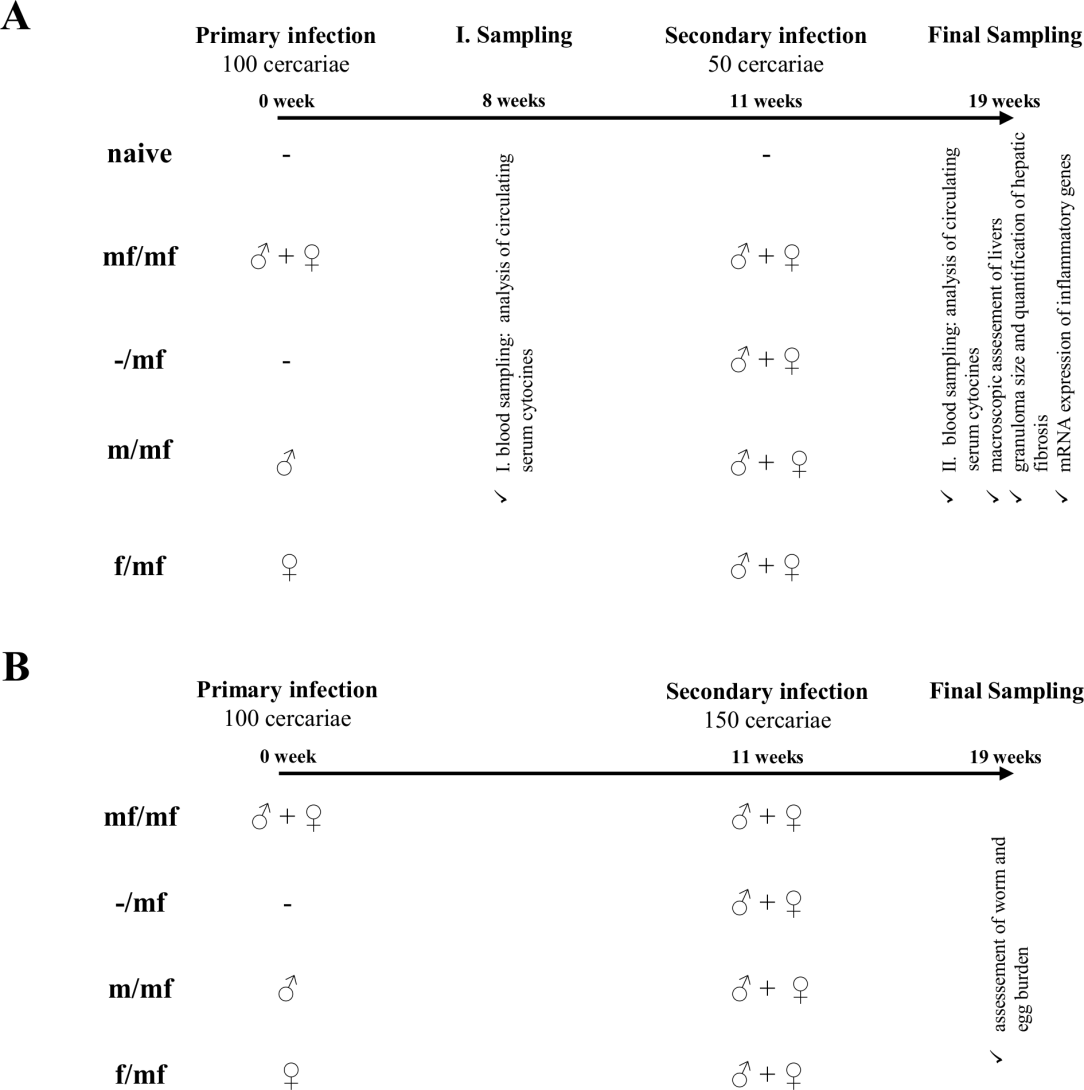
Experimental setup. To evaluate the effect of a primary unisexual *S*. *mansoni* infection on a bisexual challenge, two independent assessments were performed: **(A)** Assessment of serum cytokines and hepatic damage and **(B)** Determination of worm and egg burden.

Eleven weeks after the primary infection, mice (groups mf/mf, m/mf and f/mf) were percutaneously infected a second time with 50 *S*. *mansoni* cercariae of both sexes, and an additional control group (-/mf) infected for the first time. The naive control group was not infected. Blood was sampled for the second time and an assessment of signs of disease progression performed at week 19, when mice were sacrificed via cervical dislocation under ketamine/xylazine anesthesia (Fig 1A).

The experiments were then repeated with an infection dose of 150 *S*. *mansoni* cercariae to ensure there was a high number of adult worms and eggs in all mouse livers (Fig 1B). In the primary infection step (week 0), mice were percutaneously infected with 100 male and female (mf/mf) *S*. *mansoni* cercariae, 100 male (m/mf) or 100 female (f/mf) *S*. *mansoni* cercariae, or not infected (naive control). Blood sampling was performed for the first time at week 8.

### Protein expression analysis using Luminex

Luminex analysis was performed using ProcartaPlex™ Multiplex Immunoassay (eBioscience, Germany) according to the manufacturer’s instructions. Serum from all time points was assayed for the murine cytokines IL-4, IL-10, IL-12p70, IL-13, TNF-α and IFN-γ. The samples and standards were measured using Bio-Plex^®^ 200 System.

### Evaluation of infection and infection-related organ alterations

At week 8 a SWAP (*S*. *mansoni* adult worm soluble antigen)–in-house ELISA [23] was performed on all mice to detect immunoglobulin G (IgG) against worms.

To determine infection burden, total egg numbers were assessed by microscopically evaluation (at 100-fold magnification) in defined, weighted liver fractions (squash slides). Adult *S*. *mansoni* worm pairs were counted using the liver perfusion technique, as described elsewhere [24].

At week 19 the weights of spleens and livers were determined and expressed as ratio of the respective organ to body weight. The extent of liver damage was assessed macroscopically on the basis of infection-related changes in liver color, stiffness and the prevalence of nodules compared to healthy controls.

For histological evaluation one half of the right liver lobe was fixed in 10% neutral buffered formalin solution (Sigma Aldrich, Germany) and embedded in paraffin. Thin sections of 5 μm were stained with either hematoxylin/eosin (HE) or Sirius red (SR). Granuloma size was determined using ImageJ software (v1.47v; National Institutes of Health, USA).

### Quantification of hepatic fibrosis

The extent of hepatic fibrosis was analyzed on the basis of thin sections stained for collagen (Sirius red, SR). The SR-positive areas were assessed using ImageJ software (v1.47v; National Institutes of Health, USA).

### RT-PCR for proinflammatory and profibrotic gene expression

Liver specimens were snap-frozen in nitrogen-cooled methylene butane and then stored in liquid nitrogen until RNA analysis. Total RNA was isolated (RNeasy Plus Mini Kit, Qiagen, Germany) and reversely transcribed into cDNA using High-capacity cDNA Reverse Transcriptase Kit (ThermoFisher, Germany) according to the manufacturer’s instructions. Real-time PCR (RT-PCR) was performed in triplicates using the following TaqMan Gene Expression Assays: Foxp3 Mm00475162; Ctla4 Mm00486849; Arg1 Mm00475988; Retnla Mm00445109 (ThermoFisher, Germany). The reaction was performed on the 7900HT Fast Real-Time PCR System under the following reaction conditions: thermal cycling conditions were 50°C for 2 minutes followed by 95°C for 10 minutes, 45 cycles at 95°C for 15 seconds, and at 60°C for 1 minute. Gene expression values were normalized to the endogenous reference gene GAPDH (Rodent GAPDH control reagent, ThermoFisher, Germany) and presented as normalized expression values relative to naive controls.

### Serum biochemistry

Serum biochemistry for alanine aminotransferase (ALT), aspartate aminotransferase (AST) and alkaline phosphatase (AP) was performed using UniCel® *DxC 800* Synchron® Clinical System (Beckman Coulter GmbH).

### Statistics

Statistical analysis was performed using the GraphPad Prism 4.0 (GraphPad Software, San Diego, CA). Values are expressed as mean ± standard deviation (SD). Groups were compared using ANOVA (with Tukey post-hoc) and, in the event of non-normality, using the Kruskal-Wallis test (with subsequent Mann–Whitney U-tests, pairwise). Normal distribution was checked using the Kolmogorov-Smirnov test. P≤0.05 (Bonferroni-adjusted for multiple testing) was considered to be statistically significant.

## Results

### Primary infection with female *S*. *mansoni* cercariae mitigates liver pathology and hepatic fibrosis after secondary infection

**Fig 2:**
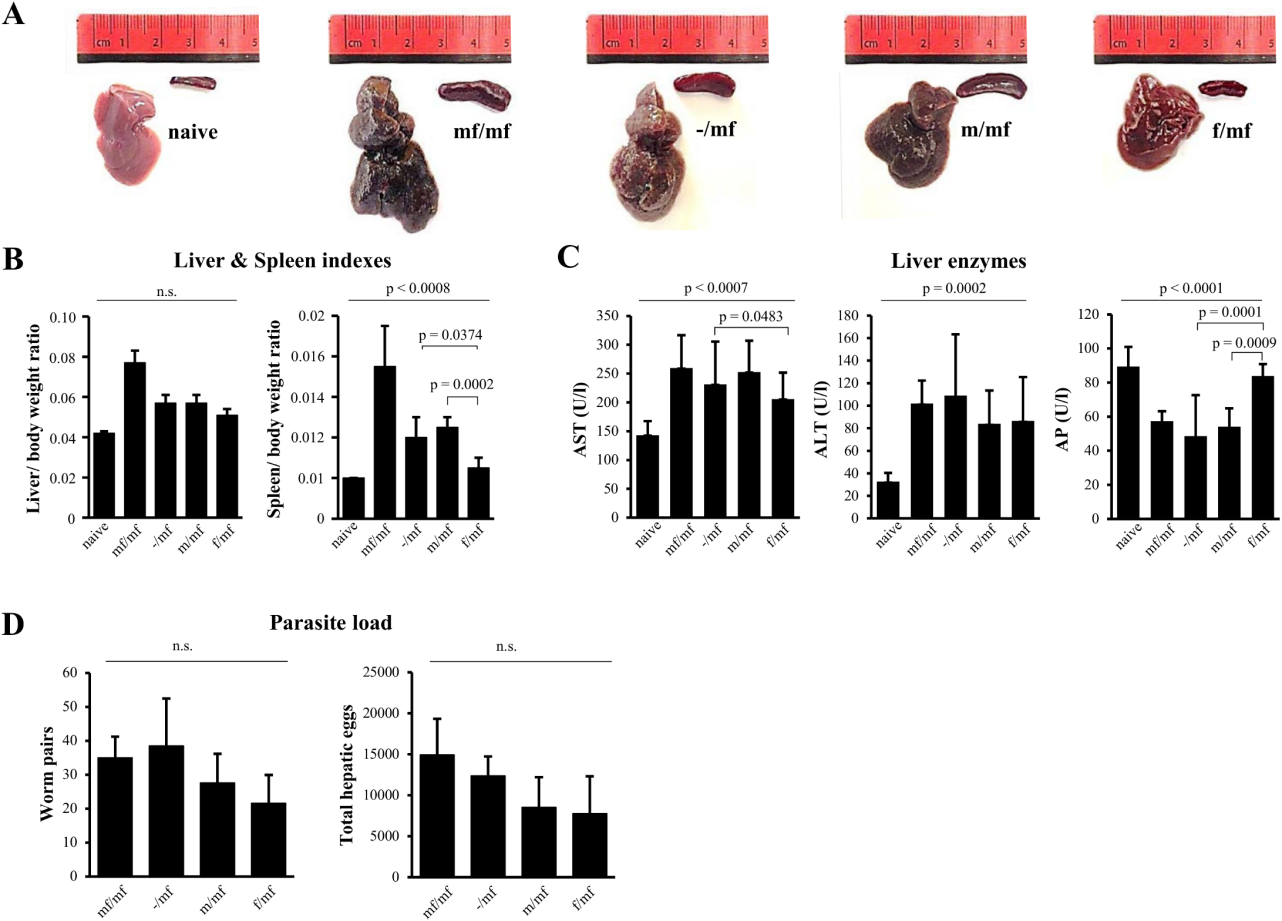
Primary infection with female *S*. *mansoni* cercariae ameliorates disease progression in bisexually challenged mice. Representative images of mouse livers and spleens are shown: uninfected mice (naive), bisexual infected control (mf/mf), infected bisexual control (-/mf), primary infection with male cercariae (m/mf), primary infection with female cercariae (f/mf). **(A)** Macroscopic assessment of livers and spleens reveals less pronounced organ changes in group f/mf than in mf/mf, -/mf and m/mf. **(B)** Reduction in liver and spleen indexes in group f/mf indicates that a primary infection with female *S*. *mansoni* cercariae has a beneficial effect on disease progression. Data are presented as mean ± SD. **(C)** Serum levels of aspartate aminotransferase (AST), alanine aminotransferase (ALT) and alkaline phosphatase (AP) are presented as mean ± SD. **(D)** Numbers of worm pairs and hepatic eggs are given as mean ± SD. P≤0.05 was considered to be statistically significant.

At week 19 macroscopic evaluation of the livers of f/mf mice revealed smooth surfaces with no macroscopically visible nodules and a mean weight of 1.28 ± 0.14 g, while the livers of m/mf mice were enlarged (1.34 ± 0.07 g) with rough surfaces and clearly visible nodules. These alterations were even more prominent in group mf/mf, which presented greyish, nodular livers and a mean liver weight of 1.92 ± 0.15 g (Fig 2A). Group f/mf had lower liver and spleen ratios than the other infected groups (Fig 2B). Serum ALT and AST levels were significantly elevated in all infected groups compared to the naive controls, while levels of AP were not (Fig 2C). Parasite load in groups f/mf and m/mf reached comparable levels (hepatic eggs: f/mf 7775 ± 4537 and m/mf 8522 ± 3668; worm pairs: f/mf 22 ± 8 and m/mf 28 ± 9, p> 0.05). The highest infection burden was detected in group mf/mf and -/mf (hepatic eggs: mf/mf 14921 ± 4401 and -/mf 12357± 2368; worm pairs: mf/mf 35 ± 6 and -/mf 39 ± 14, p> 0.05) (Fig 2D).

**Fig 3:**
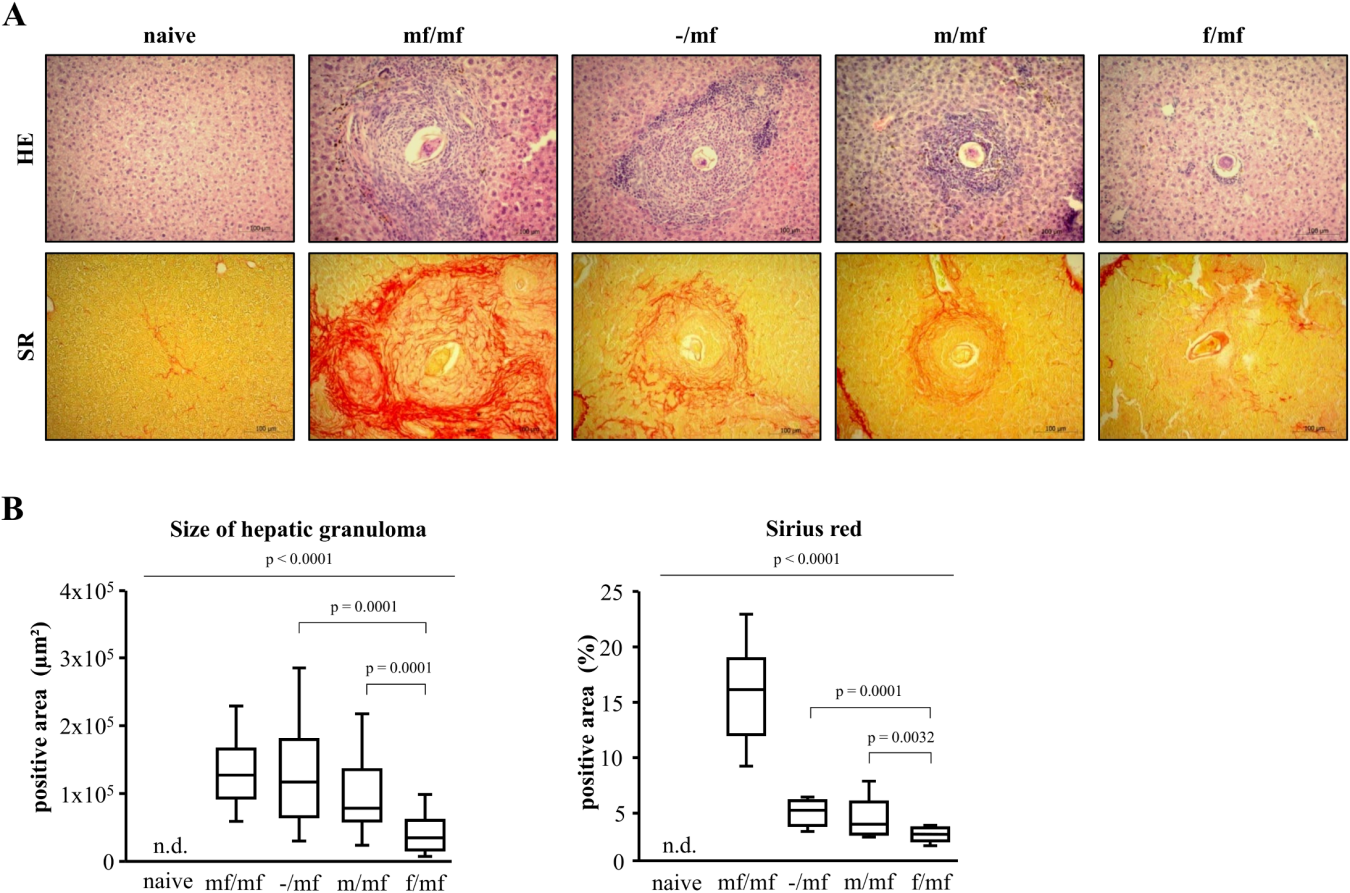
Primary infection with female *S*. *mansoni* cercariae reduces granuloma size and hepatic fibrosis in bisexually challenged mice. **(A)** Representative images of hematoxylin/eosin (HE) and Sirius Red (SR)-stained liver slices are shown (original magnification 100-fold): uninfected mice (naive), bisexual infected control (mf/mf), infected bisexual control (-/mf), primary infection with male cercariae (m/mf), primary infection with female cercariae (f/mf). Primary infection with female *S*. *mansoni* cercariae in group f/mf resulted in smaller peri-oval granulomas and reduced fibrotic peri-portal bridging compared to groups mf/mf, -/mf and m/mf. **(B)** This was quantitatively confirmed by morphometric analysis of granuloma diameter and Sirius Red stained areas. n.d., not detectable. p≤0.05 was considered to be statistically significant.

Hepatic granulomas were detectable in all infected groups (Fig 3A). Primary infection with female *S*. *mansoni* cercariae (f/mf) led to significantly smaller granulomas than in the other infected groups (Fig 3B). The largest granulomas, accompanied by a pronounced formation of fibrotic septa, were found in group mf/mf. Mice livers from the groups m/mf and -/mf displayed comparable granuloma sizes and early signs of porto-portal bridging. Quantification of Sirius red-positive areas revealed the highest collagen deposition in group mf/mf and significantly less pronounced hepatic fibrosis in group f/mf (Fig 3B).

### Single-sex infection alters TH2 response in a gender-specific manner

**Fig 4:**
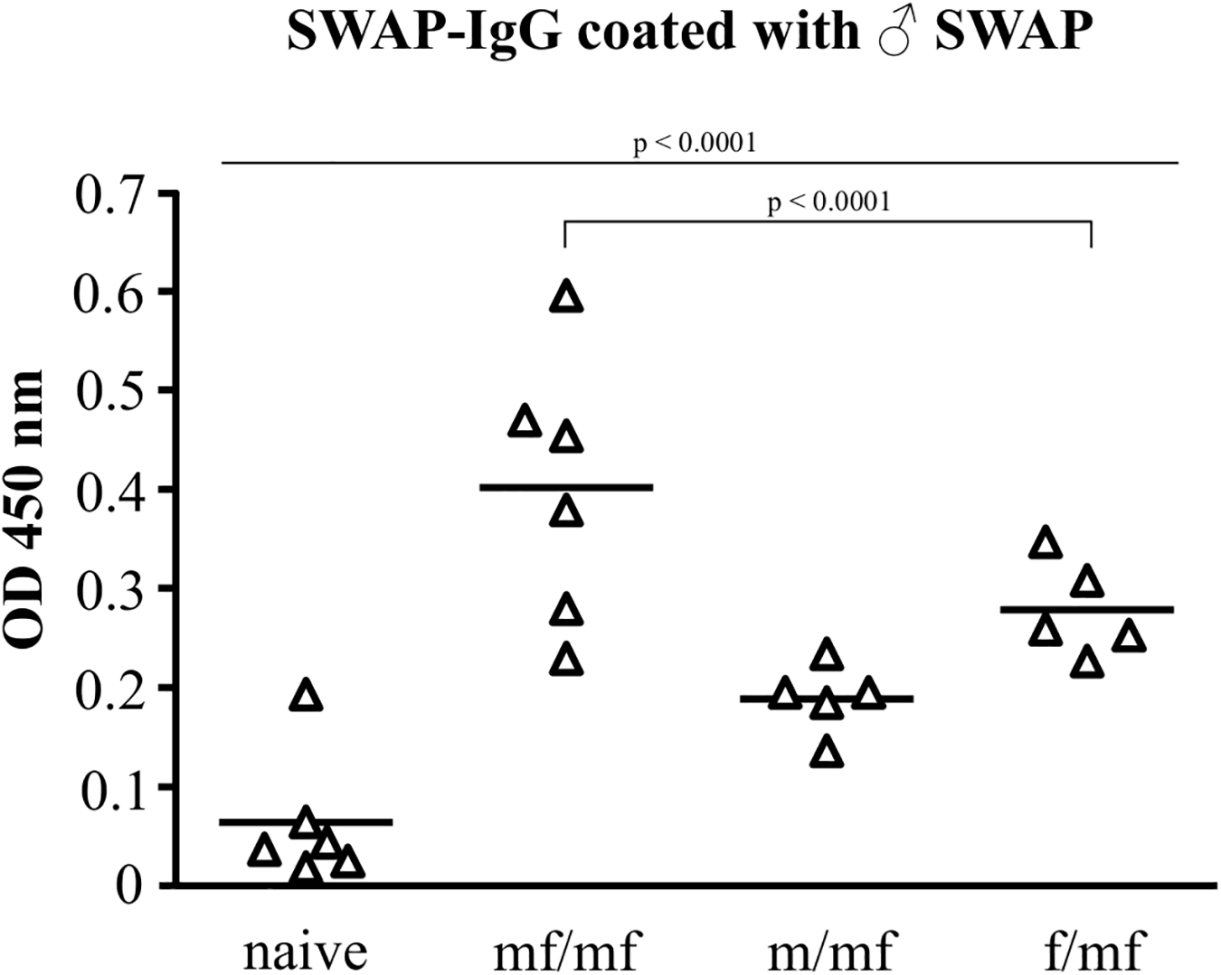
Proof of primary infection at week 8. Comparable antibody levels against male *S*. *mansoni* worm antigens (SWAP ELISA) confirm infection with 100 male (m/mf), 100 female (f/mf) or 100 male/female (mf/mf) *S*. *mansoni* cercariae. Naive mice make up the healthy control group. p≤0.05 was considered to be statistically significant.

At week 8, infection was verified using SWAP-ELISA, which detected antibodies against *S*. *mansoni* worm antigen in all infected groups (Fig 4). TH1-cytokines (TNF-α, IFN-γ, IL-12p70) and TH2-cytokines (IL-4, IL-10, IL-13) were measured 8 weeks after initial infection.

**Fig 5:**
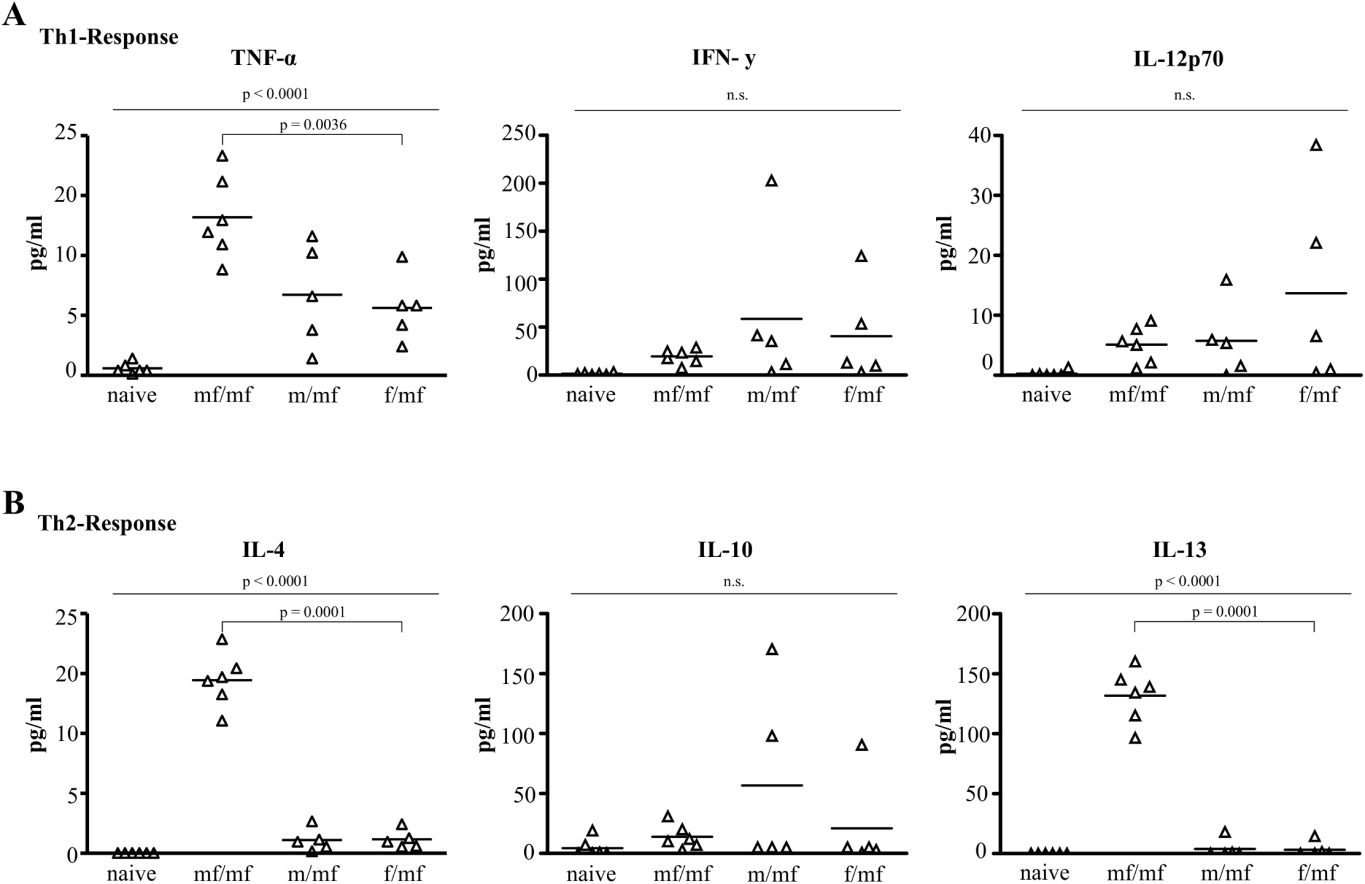
Eight weeks after primary infection, single male and single female schistosomes fail to induce a TH2 response. Standard box plot of circulating cytokine level of **(A)** TH1 and **(B)** TH2 in mouse sera analyzed using ProcartaPlex™ Multiplex Immunoassay: uninfected (naive), bisexually infected at both infection time points (mf/mf), bisexually infected at second infection time point (-/mf), primarily infected with male cercariae and bisexually challenged (m/mf), primarily infected with female cercariae and bisexually challenged (f/mf) mice. p≤0.05 was considered to be statistically significant.

TNF-α and IFN-γ serum levels were significantly higher in groups f/mf, m/mf and mf/mf than in the naive control group, with the highest levels of TNF-α found in group mf/mf (Fig 5A). This indicates a proinflammatory TH1-reaction. IL-4 and IL-13 were solely detectable in group mf/mf (Fig 5B). Levels of IL-10 did not differ between the groups (f/mf, m/mf, mf/mf, naive).

**Fig 6:**
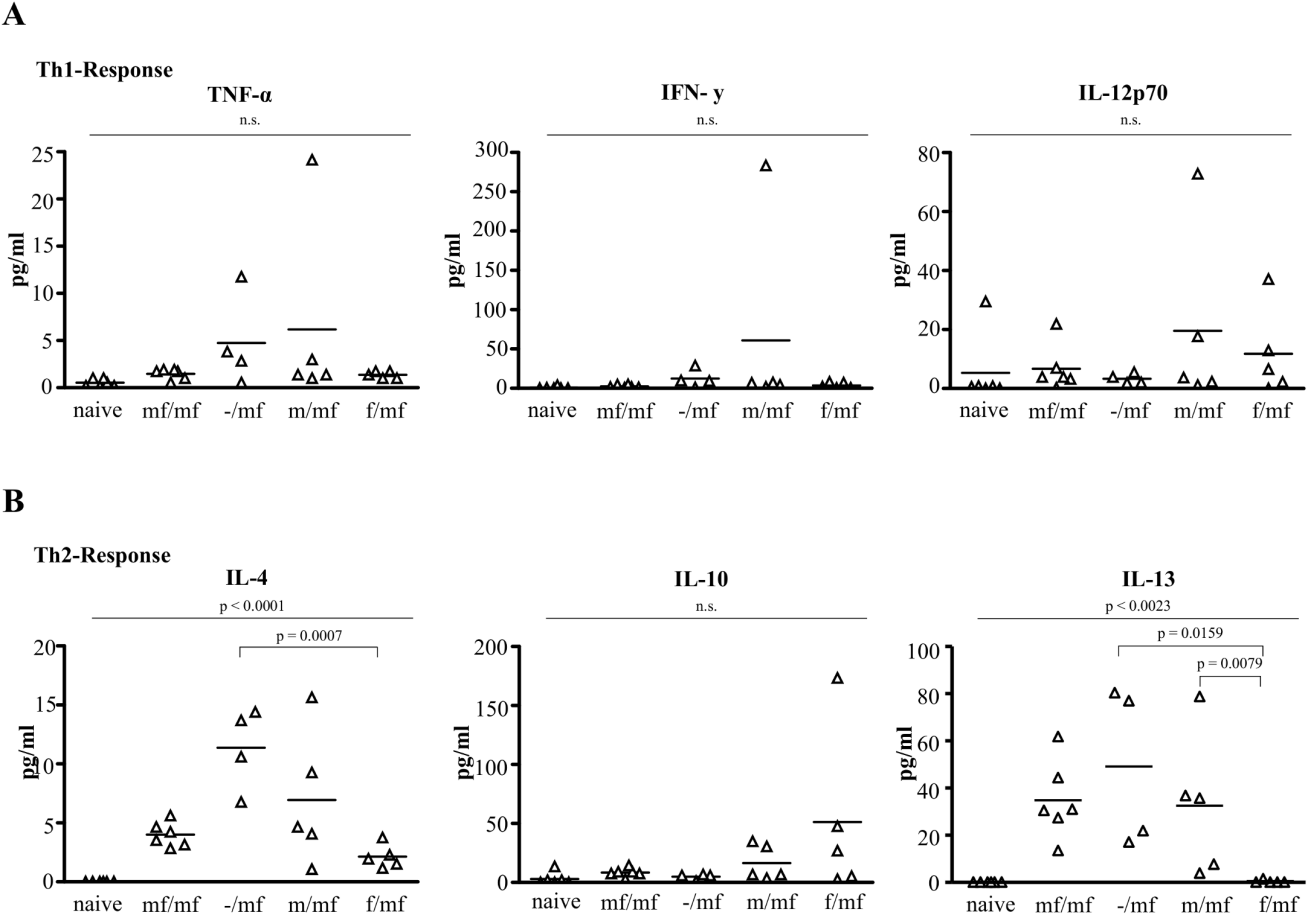
Primary infection with female *S*. *mansoni* cercariae suppresses TH2 response in bisexually challenged mice. Standard box plot of circulating cytokine level of **(A)** TH1 and **(B)** TH2 in mouse sera analyzed using ProcartaPlex™ Multiplex Immunoassay: uninfected (naive), bisexually infected at both infection time points (mf/mf), bisexually infected at second infection time point (-/mf), primarily infected with male cercariae and bisexually challenged (m/mf), primarily infected with female cercariae and bisexually challenged (f/mf) mice. p≤0.05 was considered to be statistically significant.

At week 19 the numbers of surviving mice per group were: naive = 6 of 6, f/mf = 5 of 6, m/mf = 5 of 6, mf/mf = 6 of 6 and -/mf = 4 of 6. Groups f/mf, m/mf, mf/mf and -/mf displayed comparable levels of TNF-α and IFN-γ that were higher than those in the naive controls, indicating that a second infection does not influence TH1-response (Fig 6A). Expression of the TH2 cytokines IL-13 and IL-4 was lower in group f/mf than in groups m/mf, mf/mf and -/mf (Fig 6B).

### Primary infection with female *S*. *mansoni* cercariae induces Ctla4 expression in bisexually challenged mice

**Fig 7:**
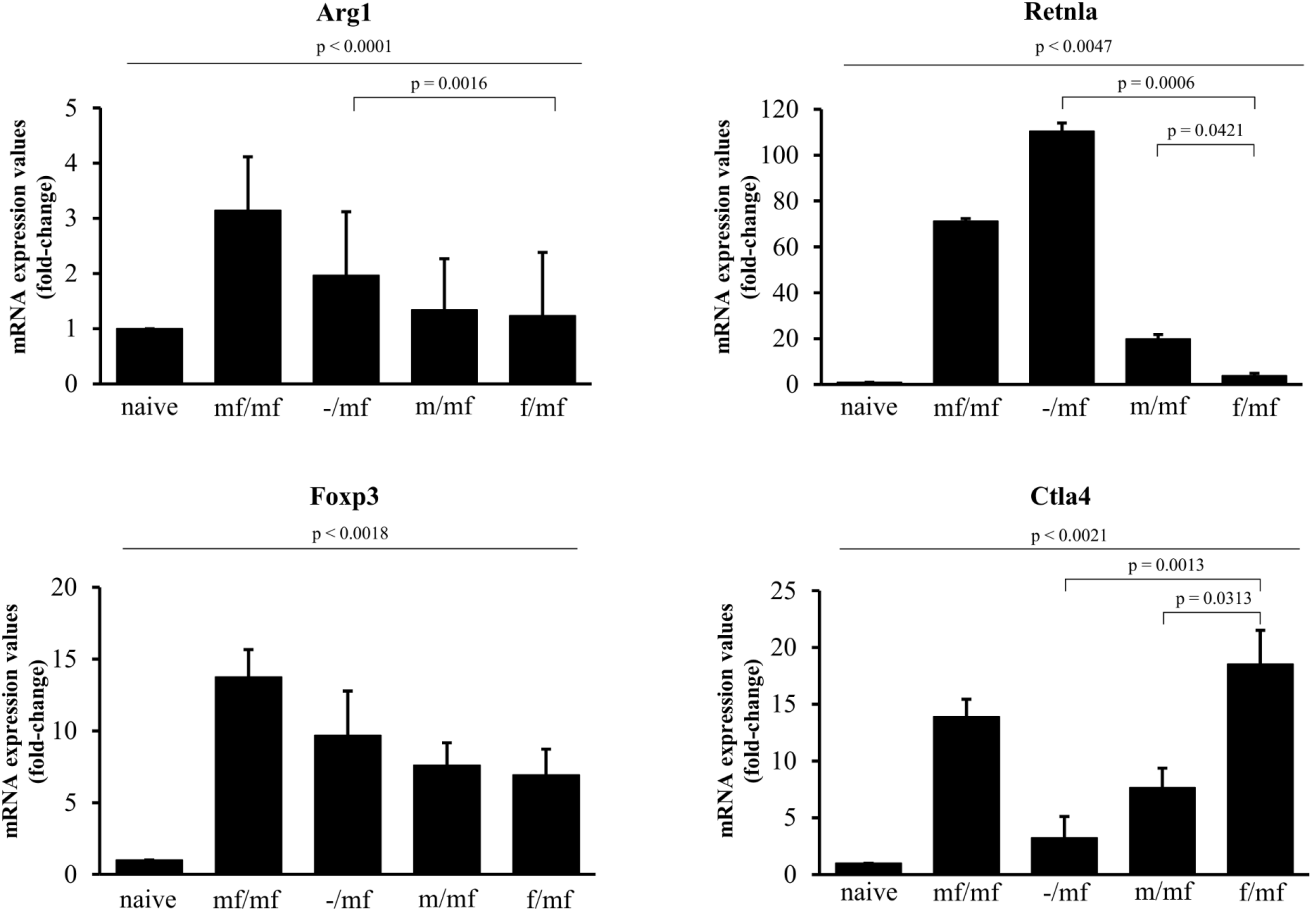
Primary infection with female *S*. *mansoni* cercariae induces Ctla4 expression in bisexually challenged mice. Gene expression values of Arg1, Retnla, Foxp3 and Ctla4 at week 19 were normalized to the endogenous reference gene GAPDH and presented as relative expression values to the naive control. Values are given as mean ± SD. p≤0.05 was considered to be statistically significant.

Arg1 and Retnla mRNA expression was measured in order to detect potential disturbances in TH2 polarization. Arg1 was expressed in all infected groups, with the highest values found in group mf/mf. Retnla expression was significantly lower in groups f/mf and m/mf than in -/mf and mf/mf. Foxp3 expression was measured in order to detect the presence of Foxp3 positive regulatory T-cells (Tregs). Foxp3 expression was highest in group mf/mf, while no differences were found between the other infected groups. Ctla4 expression, which inhibits TH2 response, was higher in group f/mf than in m/mf and -/mf (Fig 7).

## Discussion

We demonstrated that primary single-sex infection with female but not male *S*. *mansoni* cercariae decreases granuloma size and hepatic fibrosis, and is accompanied by a Ctla4-mediated suppression of TH2 hyperreactivity.

There are two basic options for reducing egg-induced pathology during *Schistosoma spp*. infection: 1) achieve resistance to reinfection, or 2) dampen down granulomatous hyperreactivity [4]. The notion that infection with different developmental stages of *Schistosoma spp*. could have an immunizing effect was based on the observation in both human and animal models that recurrent *Schistosoma spp*. infections or repeated Praziquantel treatment lead to the development of resistance to reinfection [15, 25–28]. In our study, a primary infection with male and female or exclusively male or exclusively female cercariae 11 weeks prior to a bisexual challenge did not induce resistance to reinfection, as demonstrated by comparable numbers of worm pairs and hepatic eggs. Previous studies, however, have shown that after a prior bisexual infection with *S*. *japonicum*, mice become significantly resistant to reinfection within 6 weeks, and that this resistance peaks at 8 weeks [17]. In yet another study, mice harboring light male infections of the Philippine strain of *S*. *japonicum* were not resistant when challenged 7 and 10 weeks after infection [29].

In our study, contrary to expectations, slightly higher numbers of worm pairs were detected at week 19 in group -/mf than in the doubly infected group mf/mf. We assume that worms stemming from the first infection (19 weeks) in group mf/mf may be located deeper within the mesenteric veins and could therefore be more difficult to retrieve using liver perfusion techniques. A de facto higher worm load in group mf/mf is most probable since hepatic egg load in this group exceeds numbers compared to the other groups.

Although parasite burden was not influenced by a primary infection, TH2-associated liver damage was significantly less in mice pre-infected with female *S*. *mansoni* cercariae. Besides displaying smaller hepatic granulomas and less extensive hepatic fibrosis, group f/mf also had the lowest liver and spleen indexes, indicating a slower rate of disease progression.

In contrast to our results, a study from 1997 found no difference between the size of perioval granulomas within the lungs of mice subject to prior male single-sex infection and those in mice subject to prior female single-sex infection when *S*. *mansoni* eggs were injected intravenously nine weeks later [13]. Since both the infection steps in our experiment were percutaneous, the involvement of specific worm antibodies in the modulation of immunopathology cannot be ruled out, but comparable IgG titers in all infected groups speak against this hypothesis.

In accordance with the smaller granuloma size and less extensive hepatic fibrosis in group f/mf at week 19, lower serum cytokine levels of pro-fibrotic IL-4 and IL-13 were measured. A combined knock-out of IL-4 and IL-13 leads to an increase in TH1 regulated inflammation accompanied by necrotic tissue destruction and higher mortality [30, 31]. However, we did not observe higher levels of TH1 regulated inflammation in group f/mf in our setting. In addition, the expression of Arg1 in the livers of all infected mice confirmed an adequate TH2 response. This ties in with our previous findings that in murine Schistosomiasis, bile acid treatment mediates a reduction in TH2 response and hepatic fibrosis without augmenting TH1 response [32]. TH1 and TH2 responses have been shown to be reciprocally regulated to a certain degree [33, 34]. In our experiments, however, TH1 and TH2 responses seem to be regulated independently.

The deposition of *S*. *mansoni* hemozoin pigment in mouse livers is associated with the presence of Arg1-positive macrophages that specifically lack Retnla (also known as Relmα and Fizz1) [35]. Retnla is known to be involved in the downmodulation of TH2 response (via a negative feedback regulation) [36]. In our experiments Retnla was significantly lower in group f/mf exclusively.

Unmated female schistosomes from unisexual infections are developmentally stunted and do not enter mesenteric veins as unmated male schistosomes or worm pairs do [37]. On the basis of our own and others' observations, it seems that the majority of virgin female worms are entrapped within the liver [38]. This might result in larger amounts of hemozoin pigment within the liver, accompanied by a Retnla-mediated suppression of TH2 response.

During Schistosoma infection Foxp3 positive T cells (Tregs) are key regulators of immune homeostasis. The cytotoxic T-lymphocyte-associated protein 4 (Ctla4, also known as protein receptor CD152) is constitutively expressed on Foxp3 positive Tregs and constitutes a further potent inhibitor of TH2 response by mediating T-cell anergy and tolerance [39]. In our setting Ctla4 was highest in group f/mf, while Foxp3 was uniformly expressed in all infected groups. As shown recently, blocking Ctla4 during the acute stage of Schistosoma infection results in an exaggeration of TH2 response, suggesting that high levels of Ctla4 might be involved in a reduction of TH2 response and of hepatic fibrosis. Though, this suppression of initial TH2 response is not to be confused with downmodulation of TH2 in persisting infections. Downmodulation of TH2 was observed in group mf/mf and might be an explanation for the reduction of IL-4 and IL-13 levels in group mf/mf with a longer time span of egg deposition (in total 13 weeks) versus -/mf (in total 2 weeks) [40]. In addition, Foxp3 positive Tregs can produce IL-10, which also inhibits TH2 response. Due to high variation in the values within the infected groups in our setting, we cannot rule out that IL-10 did indeed play a role [41].

In conclusion, a primary infection with female *S*. *mansoni* cercariae has a protective effect on granuloma size, hepatic fibrosis and disease progression in bisexually challenged mice. This protection is potentially associated with Ctla4-mediated TH2 suppression but not with a reduction in parasite load. Our findings provide evidence that protection against egg-induced granulomatous hyperreactivity is achievable by exploiting gender-specific differences between schistosomes.
